# Role of Baduanjin exercise-based cardiac rehabilitation in coronary heart disease after percutaneous coronary intervention: A protocol for systematic review and meta-analysis of randomized controlled trials

**DOI:** 10.1097/MD.0000000000031612

**Published:** 2022-12-16

**Authors:** Xingxing Li, Quan Lin, Rongpeng Liu, Yang Wu, Zonging Fan

**Affiliations:** a Department of Cardiology, Dongfang Hospital, Beijing University of Chinese Medicine, Beijing, China.

**Keywords:** Baduanjin exercise, cardiac rehabilitation, coronary heart disease, percutaneous coronary intervention, randomized controlled trials, systematic review

## Abstract

This study aimed to explore the effects of Baduanjin-based cardiac rehabilitation on cardiac function and quality of life in patients with coronary heart disease who have undergone percutaneous coronary intervention.

**Methods:**

PubMed, the Excerpta Medica Database, the Cochrane Library, Web of Science, the Wanfang, SINOMED, the China Science and Technology Journal Database and China National Knowledge Infrastructure were searched for appropriate articles from their respective inception until March 30, 2021. Meta-analysis was conducted with the RevMan 5.3 software.

**Results:**

A total of 11 studies including 1025 patients were considered. Compared with conventional Western medicine, Baduanjin improved the left ventricular ejection fraction of patients [mean difference (MD) = 2.83, 95% confidence interval (CI) (2.05, 3.61), *P* < .00001], increased the Seattle angina questionnaire and SF-36 health survey scale scores [MD = 6.67, 95% CI (4.09, 9.26), *P* < .00001; standard mean difference  = 0.73, 95% CI (0.55, 0.91), *P* < .00001, respectively] and decreased the scores of Zung self-rating anxiety scale and self-rating depression scale [MD = –6.64, 95% CI (–7.69, –5.22), *P* < .00001; MD = –6.63, 95% CI (–7.60, –5.66), *P* < .00001, respectively].

**Conclusion:**

Our findings showed that Baduanjin exercise improved cardiac function and quality of life and alleviated patients’ anxiety and depression.

## 1. Introduction

Coronary heart disease (CHD), also called coronary artery disease, is the most common cause of death worldwide.^[1]^ Percutaneous coronary intervention (PCI) is a common therapeutic strategy that improves angina symptoms.^[2,3]^ However, during follow-up, there are still recurrences of cardiovascular events after PCI, and patients usually suffer from low-quality life.^[4]^ Cardiac rehabilitation (CR) has been advocated by numerous clinical guidelines for patients with CHD after PCI.^[5]^ Combined with routine therapy, exercise-based CR is a convenient and safe option, according to the guidelines of the American Heart Association.^[6]^ Moreover, mild to moderate intensity physical exercise has been reported to have protective effects on survival for patients post-PCI surgery.^[7]^

As a traditional Chinese exercise, Baduanjin (also called 8-Sections Brocades) combines meditation with gentle movements and has been widely practiced in China to treat diseases.^[8,9]^ Studies have showed that Baduanjin exercise as a complementary and alternative therapy for patients with cardiovascular disease improved the clinical outcome of cardiovascular patients and reduced the occurrence of adverse cardiovascular events.^[10]^ This exercise activates a sequence of natural self-regulatory/self-healing mechanisms to stimulate the balanced release of endogenous neurohormones, is easy to master in a short period of time and is suitable for all age groups.^[11]^ A growing number of randomized controlled trials (RCTs) have been carried out to explore the effects of Baduanjin exercise on the cardiac rehabilitation of patients with CHD after PCI. Regrettably, the sample sizes of these studies have been small.

Therefore, we performed a systematic review of RCTs to evaluate the effects of Baduanjin exercise on cardiac rehabilitation in patients with CHD after PCI to provide more powerful evidence for treating post-PCI CHD patients in the clinic.

## 2. Methods

We followed the methods of Li, Xing-Xing et al 2021.^[12]^

### 2.1. Study registration

The protocol was registered in PROSPERO (No. CRD42020216513). In addition, it was also prepared based on the Preferred Reporting Items for Systematic Reviews and Meta-Analyses Protocols statement guidelines.^[13]^

### 2.2. Eligibility criteria

#### 2.2.1. *Participants*.

Patients at least 18 years old with CHD who underwent a Baduanjin exercise intervention after PCI were included without limitations on race, gender, education or economic level.

#### 2.2.2. *Interventions*.

Baduanjin exercise was used for CHD patients post-PCI surgery. Included variations in intensity, frequency and duration were acceptable.

#### 2.2.3. *Control treatments*.

Patients in the control group received conventional Western medicine (CWM) or conventional exercise (CE).

#### 2.2.4. *Outcomes*.

The primary outcomes included left ventricular ejection fraction (LVEF). The secondary outcomes included the scores of the seattle angina questionnaire (SAQ), SF-36 health survey scale (SF-36), zung self-rating anxiety scale (SAS) and self-rating depression scale (SDS).

#### 2.2.5. *Study type*.

We collected all available RCTs of Baduanjin exercise-related therapies for CHD after PCI.

### 2.3. *Exclusion criteria*

The specific exclusion criteria were as follows: non-RCTs, case reports, animal experiments, research advances, reviews, expert opinions and conference articles; patients who received other exercise measures in addition to Baduanjin exercise; duplicate articles, studies with incorrect data, inconsistency or incomplete and unavailable literature.

### 2.4. Search strategy

We searched 4 English electronic databases and 3 Chinese literature databases for studies published before November 29, 2020: PubMed, the Excerpta Medica Database, Cochrane Library, Web of Science, the Wanfang database, SINOMED, China Science and Technology Journal Database and China National Knowledge Infrastructure. In addition, there was no restriction on language. Using PubMed as an example, details of the search strategy were shown in Table 1. Moreover, manual retrieval was performed on Baidu Academic, Google Academic, books, reprints, and conference materials to obtain all the materials associated with this study as comprehensively as possible. This work was expected to be completed by 2 independent reviewers and, in cases where they had a disagreement, a 3rd person was asked for advice.

**Table 1:** Search strategy in PubMed database.

Number	Search terms	Number	Search terms
#1	Baduanjin exercise	#14	PCI
#2	Baduanjin	#15	Coronary Intervention, Percutaneous
#3	Eight section brocades	#16	Revascularization, Percutaneous Coronary
#4	#1OR#2OR#3	#17	Coronary Revascularization, Percutaneous
#5	Coronary heart diseases	#18	#13OR#14OR#15OR#16 OR#17
#6	CHD	#19	Randomized controlled trial
#7	Coronary artery disease	#20	Randomized trial
#8	Angina pectoris	#21	Clinical trial
#9	Myocardial infarction	#22	Clinical study
#10	Acute coronary syndrome	#23	Controlled study
#11	Cardi*	#24	#19OR#20OR#21OR#22OR#23
#12	#5OR#6OR#7OR#8OR#9OR#10OR#11	#25	#4 AND#12 AND#18 AND#24
#13	Percutaneous coronary intervention		

CHD = coronary heart disease, PCI = percutaneous coronary intervention.

### 2.5. Data screening and extraction

All authors received specific training on items for systematic reviews and meta-analyses protocols before the implementation of the study. The review process was independently conducted by 2 authors using blinding to reduce bias. Disagreements were settled first by discussion and then by consulting a 3rd author for arbitration. RCTs that were duplicated and did not meet the eligibility criteria were removed.

The information required was as follows: basic information: title, the 1st author’s name, and year of publication; study characteristics: interventions, course of treatment, and duration; subject characteristics: sample size, gender, age, and underlying disease; and outcomes and specific adverse reactions. When there was uncertain information in the included studies, the final decision was made through discussion among team members.

### 2.6. Risk of bias and quality assessment

Two colleagues separately assessed the quality of the included RCTs via the Cochrane Collaboration tool, and in the case of disagreement, a 3rd author was asked for advice. Review items comprised random sequence generation, allocation concealment, blinding, incomplete outcome data, selective reporting and other bias. Depending on the characteristics of the included literature, the reviewers classified studies as having low, high, or unclear risk of bias.

### 2.7. Statistical analysis

All data were statistically analyzed using RevMan5.3 software from the Cochrane Collaboration. Discontinuous variables were expressed as the risk ratio with a 95% confidence interval (CI). For continuous data, if the unit or the measurement instrument was consistent, the mean difference (MD) with 95% CI is used, otherwise, the standard mean difference with a 95% CI was used. The *χ*^2^ and *I*^2^ test were used to assess heterogeneity among the included RCTs.^[14]^ A fixed-effect model was used when heterogeneity was low (*P* ≥ .05, *I*^2^ ≤ 50%). However, when heterogeneity was high (*P* < .05, *I*^2^ > 50%), we further analyzed its potential sources based on the following 3 aspects: clinical heterogeneity, methodological heterogeneity, and statistical heterogeneity.

We evaluated clinical heterogeneity 1st, and if there was obvious clinical heterogeneity, a subgroup analysis was performed. If clinical heterogeneity was manifested and subgroup analysis could not be conducted, only descriptive analysis was used. After excluding clinical and methodological heterogeneity, statistical heterogeneity was considered, and a random-effect model was used.^[15]^ Egger and Begg test were constructed to evaluate publication bias.

#### 2.7.1. *Subgroup analysis*.

Subgroup analysis was performed to reduce the clinical heterogeneity between groups.

#### 2.7.2. *Sensitivity analysis*.

We carried out the sensitivity analyses to evaluate the reliability of results. The methods included changing the type of analysis (random-effect model or fixed-effect model), eliminating each of the included studies 1 by 1 and then combining the effect quantity.

#### 2.7.3. *Dealing with missing data*.

Original authors were contacted where possible to obtain missing information. Studies that cannot be supplemented or corrected with the required information were eliminated.

#### 2.7.4. *Assessment of reporting biases*.

Egger and Begg test were conducted to evaluate the publication bias.^[15]^

### 2.8. *Ethics and dissemination*

The ethical approval was not necessary for this meta-analysis.

## 3. Results

### 3.1. Study selection

**Figure 1.**
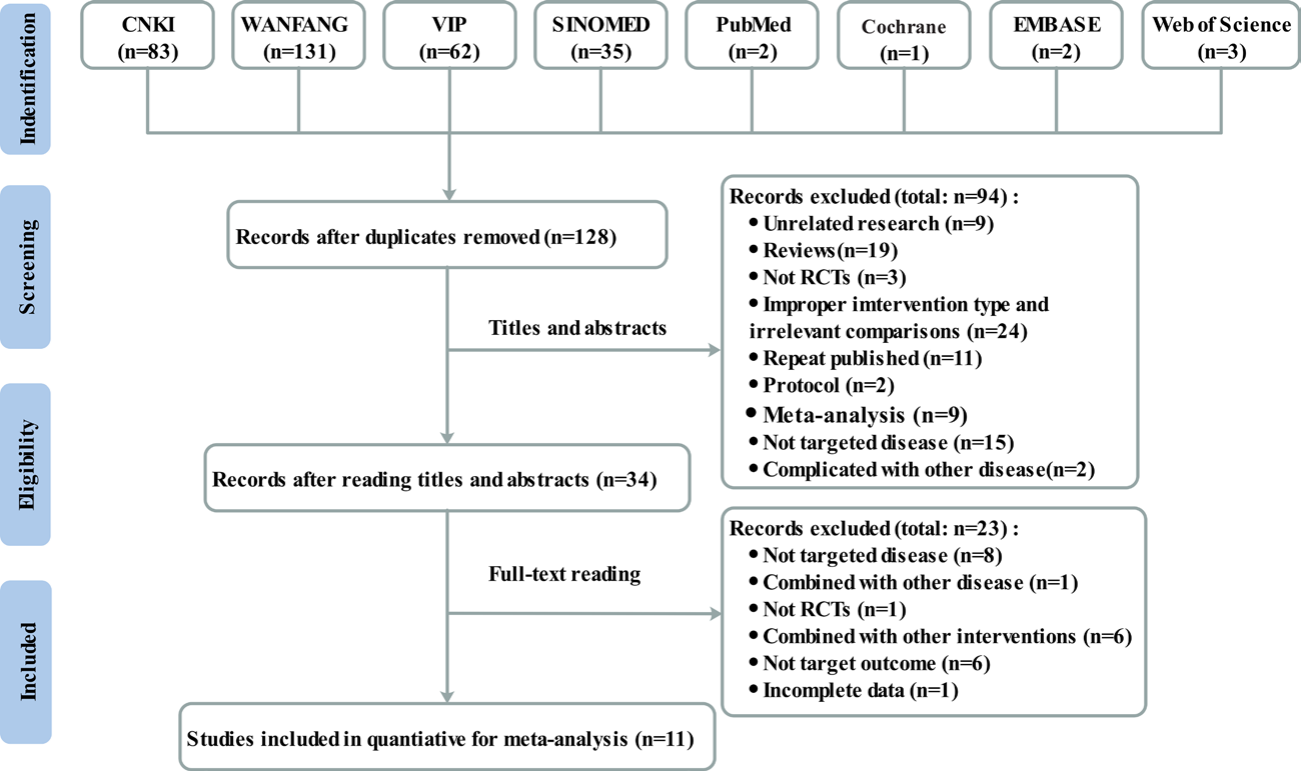
Flow diagram for study selection.

The search of 8 databases identified 319 articles for further evaluation (83 from China National Knowledge Infrastructure, 131 from Wanfang, 62 from China Science and Technology Journal Database, 35 from SINOMED, 2 from PubMed, 1 from the Cochrane Library, 2 from excerpta medica database, and 3 from the Web of Science), of which 191 were removed after reviewing due to duplicate records. After reading the titles and abstracts, 94 were excluded for various reasons. Finally, only 11 studies met our inclusion criteria after screening full texts. Figure 1 shows the detailed process of study selection process.

### 3.2. Characteristics of included studies

Eleven RCTs^[16–26]^ involving 1025 participants and performed in China from 2017 to 2020 were included in this study. The average age of the patients ranged from 47.7 to 69.64 years old. Most of the trials had more males than females. All included patients were diagnosed with CHD and successfully underwent PCI according to guidelines. All studies claimed that the baseline characteristics were comparable between groups. In 4 studies,^[23–26]^ the control group used CWM plus CE. The remaining studies^[16–22]^ were designed to compare a Baduanjin plus CWM group with a group treated with the same CWM alone. Of the 11 studies, 6 reported LVEF, 4 reported the SAQ score, 3 reported the SF-36 score, 3 reported the SAS score, 2 reported the SDS score, and only 1 reported adverse effects. The duration of the intervention ranged from 1 to 24 weeks. CWM mainly consisted of aspirin, clopidogrel, angiotensin-converting inhibitor or angiotensin II receptor blocker, *β*-blocker and statins. The dosage and type of CWM allowed the guidelines for CHD. Characteristics of the included studies are listed in table 2.

**Table 2:** Characteristics of included studies and risk of bias summary.

Study ID	Sample size (n)		Sex (M/F, n)		Age (yr)		Type of CHD	Intervention		Baduanjin Training			Outcomes
	T	C	T	C	T	C		T	C	Frequency (weekly)	Time (min)	Duration (wk)	
Chen MG et al 2020	43	39	29/14	30/9	61.49 ± 11.54	61.49 ± 11.54	MI	BE + CWM	CWM	5	30	24	LVEF, SF-36
Gu F et al 2018	50	50	35/15	39/11	59.42 ± 8.99	59.32 ± 10.02	NR	BE + CWM	CWM	3-5	NR	12	SAQ
Hu L 2018	40	40	22/18	24/16	47.7 ± 9.12	47.75 ± 9.89	NR	BE + CWM	CWM	3	60	8	SAQ, SAS, SDS
Hua L et al 2018	60	60	36/24	34/26	17 (<60) 24 (60~69) 19 (≥70)	12 (<60) 27 (60~69) 21 (≥70)	NR	BE + CWM	CWM	14	30	1	SAS
Li YS et al 2018	53	53	28/25	30/23	61.27 ± 10.39	61.38 ± 10.21	MI	BE + CWM	CWM	6	40	4	LVEF
Peng JY 2017	28	29	20/8	20/9	69.64 ± 9.25	70.5 ± 9.695	AP、MI	BE + CWM	CWM	5	30	4	SF-36
Tang T et al 2019	46	47	37/9	38/9	60.02 ± 8.66	61.38 ± 9.21	NR	BE + CWM	CWM	5	38	12	LVEF
Wang JM et al 2018	75	75	53/22	53/22	59.3 ± 15.4	58.8 ± 12.5	MI	BE + CWM + CE	CWM + CE	3-5	40	24	LVEF
Wang JJ et al 2019	55	55	27/28	28/27	58.32 ± 9.74	60.32 ± 7.23	NR	BE + CWM + CE	CWM + CE	5	30	20	LVEF, SAS, SDS
Wang XJ et al 2019	30	30	18/12	21/9	60.11 ± 8.54	59.51 ± 8.93	NR	BE + CWM + CE	CWM + CE	3-4	20	1	SAQ, SF-36
Zhang ZL 2019	33	34	21/12	23/11	59.42 ± 7.022	58.65 ± 7.027	MI	BE + CWM + CE	CWM + CE	5	40	8	LVEF, SAQ

ACS = acute coronary syndrome, AP = angina pectoris, BE = Baduanjin exercise, C = control group, CE = conventional exercise, CWM = conventional Western medicine, F = female, HE = health education, LVEF = left ventricular ejection fraction, M = male, MI = myocardial infarction, NR = not report, SAS = self-rating anxiety scale, SDS = self-rating depression scale, SAQ = seattle angina questionnaire, T = treatment group.

### 3.3. Methodological quality

**Figure 2.**
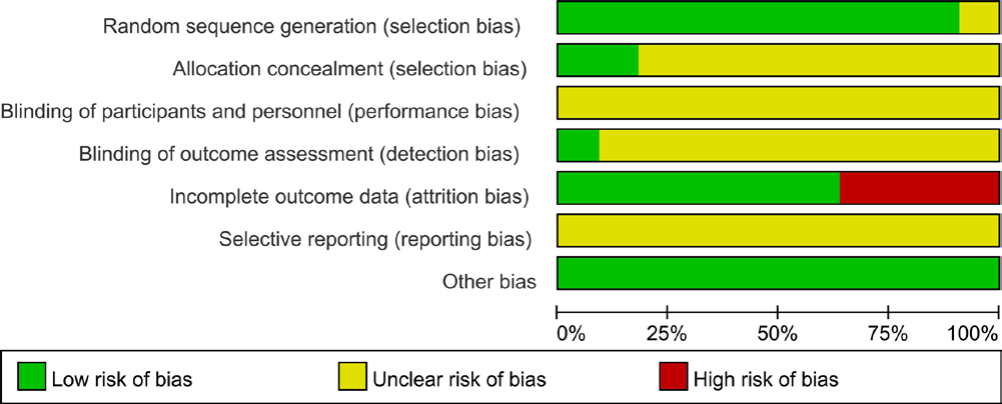
Risk of bias graph.

**Figure 3.**
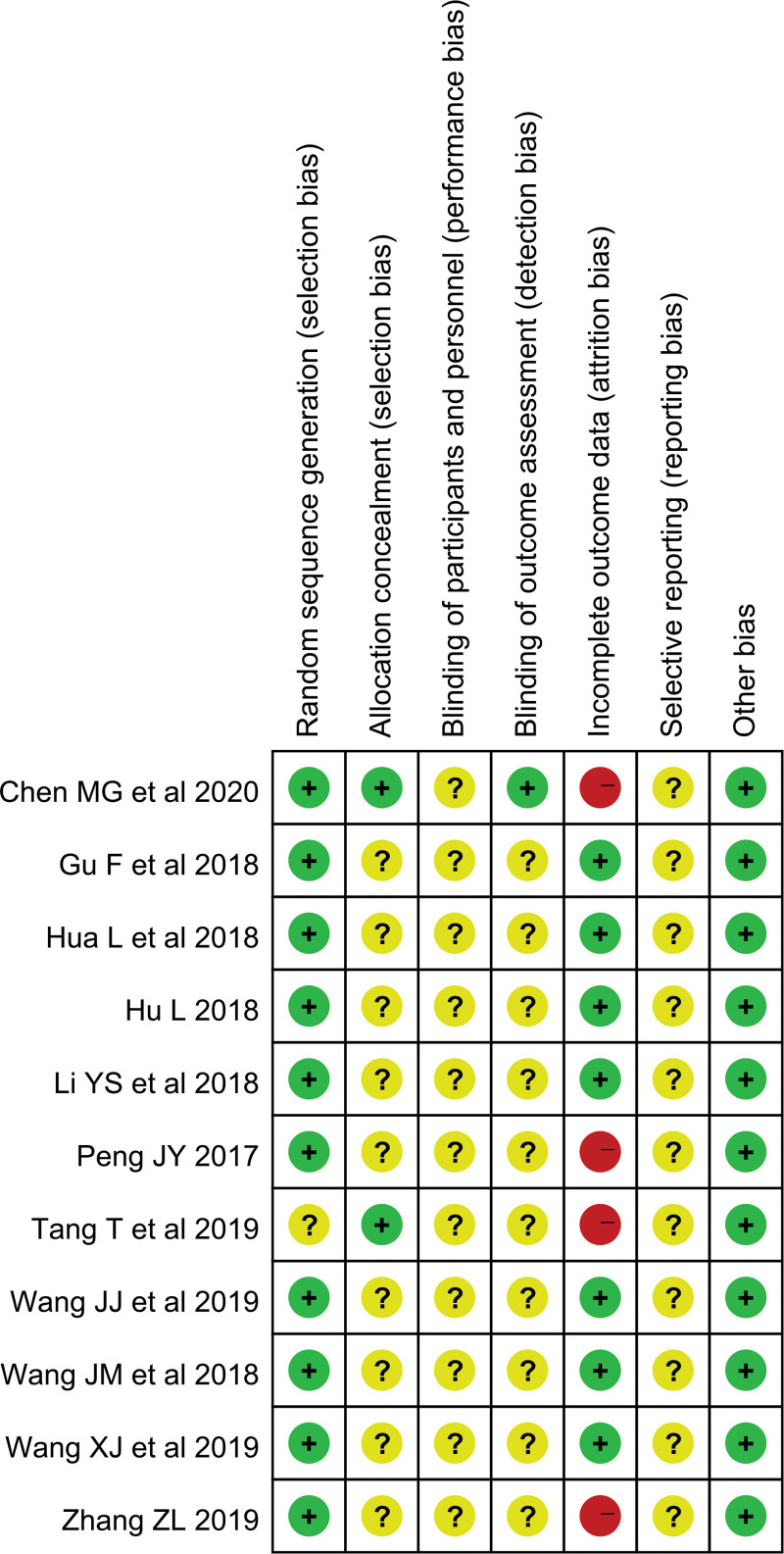
Risk of bias summary.

Figures 2 and 3 show an overview of the risk of bias for the included RCTs based on the Cochrane risk of bias tool. Ten studies were considered to have a low risk of bias in generating random sequences. In particular, 3 RCTs^[16–18]^ used a computer to generate random sequences, 7 RCTs^[19–21,24–26]^ adopted a random number table, and 1 RCT^[23]^ used random sampling. The remaining RCT^[22]^ only mentioned “randomly” in their full text but did not describe the specific methods used for random sequence generation and was therefore assessed to have an unclear risk of bias in this item. Most of the RCTs were assessed as having an unclear risk of bias on allocation concealment,^[16,22]^ which had a low risk of bias because of the use of envelope concealment. All studies were reviewed as having an unclear risk of bias on performance bias and selective reporting. Only 1 study^[16]^ applied blinding to the outcome assessment and was therefore assessed as having a low risk of bias. The other 10 RCTs^[17–26]^ were evaluated as having an unclear risk bias because of the lack of information available to determine the risk. Four RCTs^[16,21,22,26]^ reported dropout patients and explained the reason, however, they did not use intention-to-treat analysis to process missing data, and therefore, the risk of bias of reporting incomplete outcome data was judged as high. The remaining 7 RCTs^[17–20,23–25]^ claimed that there was no dropout and were evaluated as having a low risk of bias. All RCTs were evaluated as having a low risk of other bias because they reported that the baseline characteristics were comparable between the groups.

### 3.4. Outcome measures

#### 3.4.1. *LVEF*.

**Figure 4.**
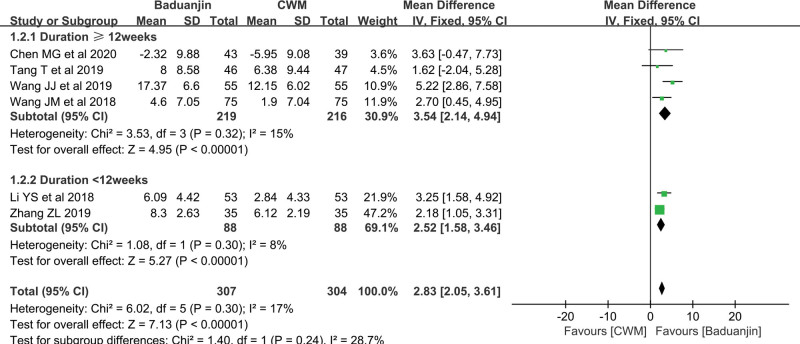
Forest Plot of Improvement on LVEF. LVEF = left ventricular ejection fraction.

Six studies reported that LVEF values were divided into subgroups corresponding to the duration of intervention of ≥ 12 weeks and < 12 weeks. For interventions with a duration ≥ 12 weeks, the results of 4 of the studies suggested that the Baduanjin group achieved a greater improvement (MD = 3.54, 95% CI [2.14, 4.94], *P* < .00001) than the CWM group, with low heterogeneity (I^2^ = 15%, *P* = .32) (Fig. 4). For interventions with a duration of < 12 weeks, the results of 2 of the studies also showed that Baduanjin plus CWM significantly improve LVEF (MD = 2.52, 95% CI [1.58, 3.46], *P* < .00001), with low heterogeneity (I^2^ = 8%, *P* = .30) (Fig. 4). All the above results showed that Baduanjin improved the cardiac function of patients who received PCI.

#### 3.4.2. *SAQ*.

**Figure 5.**
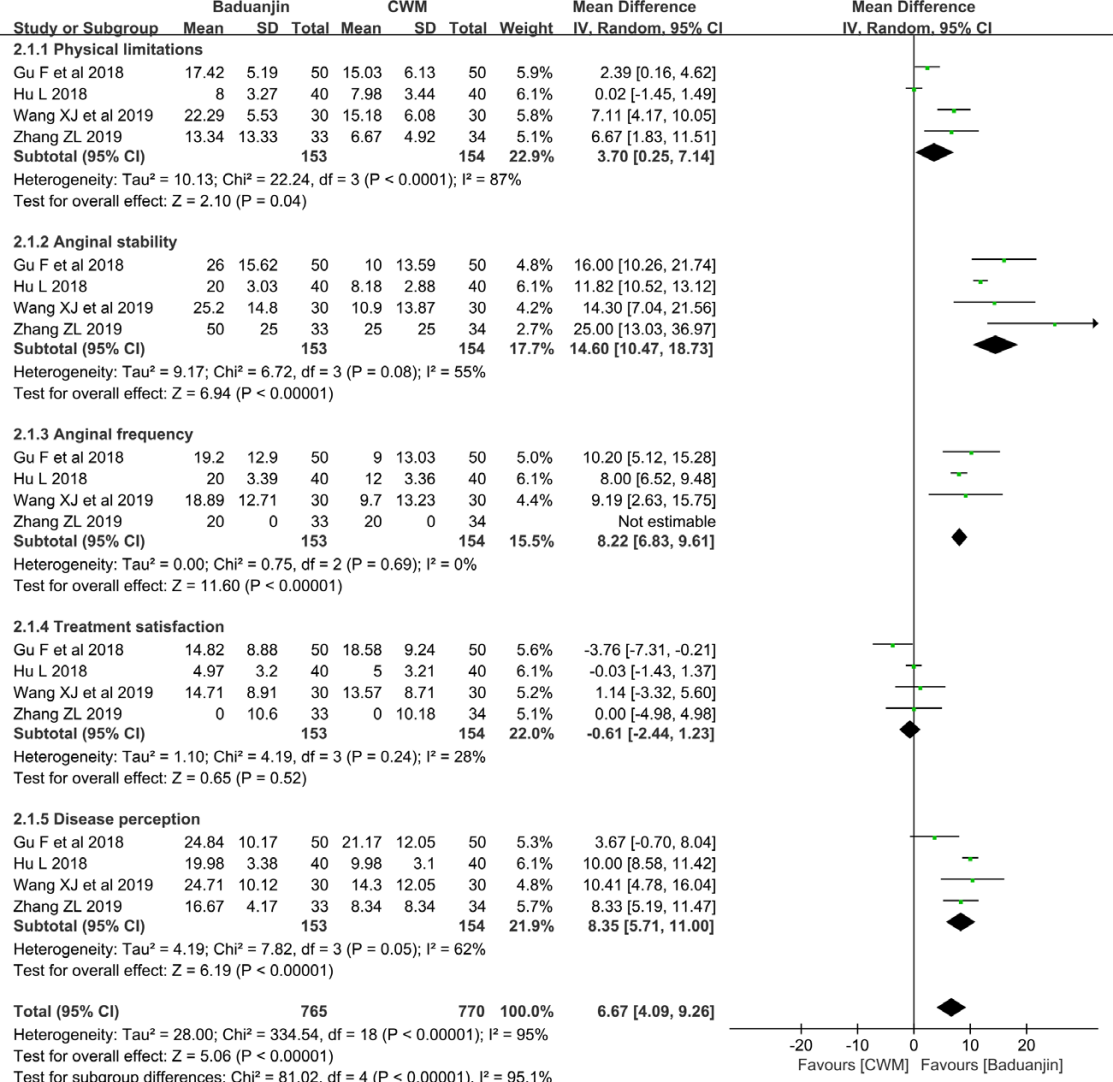
Forest Plot of Improvement on SAQ. SAQ = seattle angina questionnaire.

The SAQ scores were reported in 4 studies as follows: 153 in the Baduanjin group and 154 in the CWM group. The SAQ was divided into 5 subscales: physical limitations, anginal stability, anginal frequency, treatment satisfaction and disease perception. A higher score indicates improved quality of life. In this study, the SAQ scores were presented as different values (posttreatment–pretreatment). As shown in Figure 5, the total meta-analysis showed that the Baduanjin group achieved more improvement (MD = 6.67, 95% CI [4.09, 9.26], *P* < .00001) than the CWM group. Consistently, based on the SAQ, physical limitations, anginal stability, anginal frequency, and disease perception were significantly different between the 2 groups, with differences of 3.70 (95% CI [0.25, 7.14], *P* = .04) in physical limitations, 14.6 (95% CI [10.47, 18.73], *P* < .00001) in anginal stability, 8.22 (95% CI [6.83, 9.61], *P* < .00001) in anginal frequency and 8.35 (95% CI [5.71, 11.00], *P* < .00001) in disease perception. However, there was no obvious difference in treatment satisfaction (MD = –0.61, 95% CI [–2.44, 1.23], *P* = .52). High or low heterogeneity was found between the Baduanjin and the CWM groups in addition to anginal frequency.

#### 3.4.3. *SF-36*.

**Figure 6.**
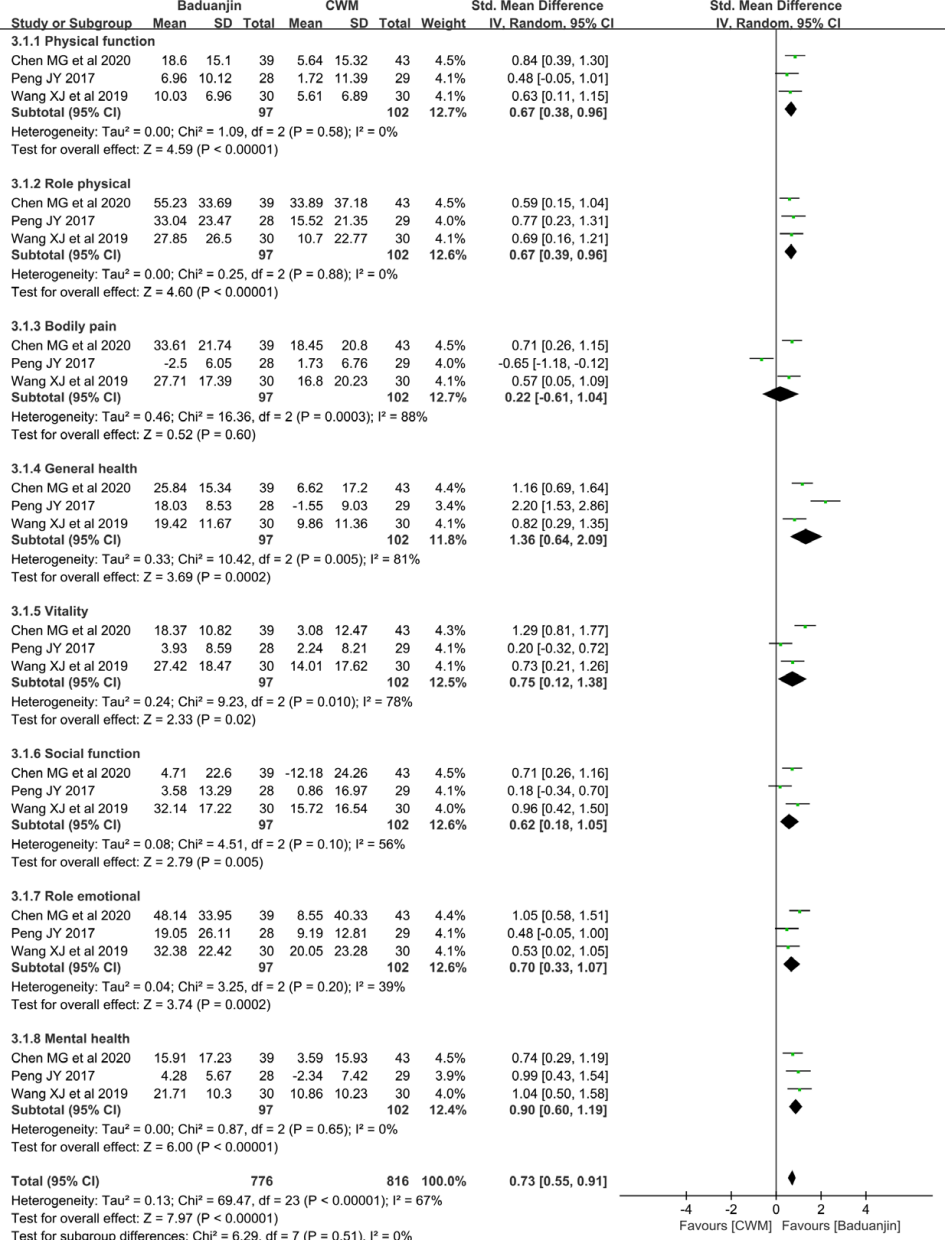
Forest Plot of Improvement on SF-36. SF-36 = SF-36 health survey scale.

Three studies provided data the SF-36 scores, and the results showed that in patients with CHD after PCI, the improvement in the SF-36 score was better with Baduanjin plus CWM/CWM + CE than with CWM plus CE or CWM alone (standard mean difference = 0.73, 95% CI [0.55, 1.19], *P* < .00001) (Fig. 6). SF-36 includes the following 8 subscales: physical function, role physical, bodily pain, general health, vitality, social function, role emotional and mental health, with a higher score indicating a better quality of life. The scores in this study were expressed as an increase in value between post- and pretreatment. In detail, Baduanjin combined with CWM/CWM + CE was better than CWM plus CE or CWM alone in terms of physical function, role physical, general health, vitality, social function, role emotional and mental health [0.67 (95% CI (0.38, 0.96), *P* < .00001], 0.67 [95% CI (0.39, 0.96), *P < *.00001], 1.36 [95% CI (0.64, 2.09), *P* = .0002], 0.75 [95% CI (0.12, 1.38), *P* = .02], 0.62 [95% CI (0.18, 1.05), *P* = .005], 0.70[95% CI (0.33, 1.07), *P* = .0002], 0.90[95% CI (0.60, 1.19), *P* < .00001), respectively] (Fig. 6). However, a totally different result was obtained for the bodily pain subscale [MD = 0.22, 95% CI (-0.61, 1.04), *P* = .60] (Fig. 6). No source of heterogeneity was found, except for bodily pain, general health, vitality, social function and role emotional.

#### 3.4.4. *SAS and SDS*.

**Figure 7.**
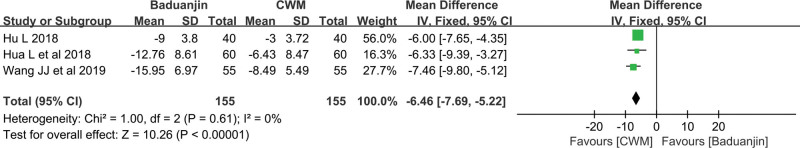
Forest Plot of Improvement on SAS. SAS = zung self-rating anxiety scale.

**Figure 8.**
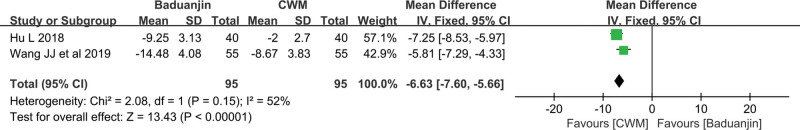
Forest Plot of Improvement on SDS. SDS = self-rating depression scale.

Three studies reported SAS scores and found a significant difference [MD = –6.46, 95% CI (–7.69, –5.22), *P* < .00001] between the Baduanjin and CWM groups. No heterogeneity was found among these studies (I^2^ = 0%, *P* = .61, Fig. 7). SDS scores wre analyzed in 2 studies including 190 patients, with 95 patients in Baduanjin group and 105 cases in CWM group. Meta-analysis showed that BE demonstrated a higher efficacy in reducing the degree of depression than CWM [MD = –6.63, 95% CI (–7.60, –5.66), *P* < .00001] (Fig. 8).

#### 3.4.5. *Adverse events*.

Only 1 study reported adverse events during study period. One patient in the CWM group died of cardiac arrest during hospitalization, and 1 patient in the Baduanjin group withdrew from the study because of a recent diagnosis of non-Hodgkin’s lymphoma during follow-up. These adverse events were not related to the interventions.

### 3.5. *Sensitivity analyses*

Sensitivity analysis was conducted to evaluate the robustness of our findings. There are 2 ways of sensitivity analysis, which include changing the model or eliminating individual studies 1 by 1. The results demonstrated that the effect values were consistently stable, indicating that the results of our study were relatively stable.

### 3.6. Publication bias

Except for SAQ, other outcomes did not show significant publication bias (LVEF: *P* = .334 for Egger test and *P* = .462 for Begg test; SF-36: *P* = .181 for Egger test and *P* = .368 for Begg test; SAS: *P* = .201 for Egger test and *P* = .296 for Begg test; SDS: *P* = 1.000 for Begg test).

## 4. Discussion

To our knowledge, this is the first systematic review and meta-analysis to explore the potential of Baduanjin as a complementary and alternative CR for patients with CHD after PCI in terms of improving cardiac function, quality of life and psychological status. Eleven RCTs involving 1025 patients were included in this study. Our findings provided objective evidence that Baduanjin exercise showed benefits in improving cardiac function and quality of life of patients with CHD after PCI compared to CWM. Moreover, combination therapy (Baduanjin combined with CE) was superior to CE. No increased risk of adverse events was associated with Baduanjin exercise. Thus, these findings supported the applicability of Baduanjin in CHD patients after PCI. However, some heterogeneity existed in this systematic review, which was related to the training intensity and duration of Baduanjin and to a low quality of studies.

Baduanjin exercise improved cardiac function. Most patients with CHD usually had cardiac dysfunction^.[27]^ LVEF is a common parameter for the assessment of cardiac function.^[28]^ The results of this study suggested that LVEF in the Baduanjin group was higher than that in the CWM group, and there was a statistical difference, which was consistent with the results of Gai TT et al^[29]^ Baduanjin is an aerobic exercise with a low intensity. It has been demonstrated that regular aerobic exercise changed the myocardial microstructure, reduced myocardial fibers, improved the elasticity of the valve, enhanced sympathetic excitability, and increased catecholamines in the blood, thereby enhancing myocardial contractility and improving the heart’s pumping capacity.^[29,30]^ Therefore, Baduanjin should be developed as a potential method for the treatment of CHD.

Baduanjin exercise improved the quality of life. Patients with CHD usually experience impaired quality of life.^[31]^ Baduanjin exercise is of great benefit to people’s health and heart-related diseases and is suitable for any population, as well as for different ages. It is also low risk and be used to improve the quality of life of CHD patients.^[32]^ Baduanjin improved the quality of life by increasing the SAQ and SF-36 scores. These results suggested the value of Bduanjin in CHD treatment. Patients from the postintervention group were most likely to have a better health-related knowledge, mastered self-care skills, and increased daily activities to improve their cardiac function, quality of life and prognosis.

Baduanjin exercise alleviated patients’ negative emotions. There are data suggesting that approximately 40% of CHD patients suffer from anxiety and depression.^[33]^ Psychological factors such as anxiety and depression are prognostic adverse factors in patients with CHD.^[34,35]^This study showed that Baduanjin exercise was associated with the improvement in the symptoms of anxiety and depression in patients with CHD after PCI. Baduanjin emphasizes the mind-body integration and is considered an effective exercise for health promotion.^[36]^ With symmetrical body postures and movements, breathing control, a meditative state of mind and mental focus, bad emotions, such as anxiety and depression, are alleviated and released, which plays a positive role in the mental health of patients.^[37]^ Moreover, numerous studies in both healthy and disease-based populations have shown that meditation improves anxiety, depression, or overall well-being.^[38]^

There were several limitations to this meta-analysis. First, unexplained high heterogeneity might have affected the results of the subgroup meta-analysis, although strict inclusion criteria were applied to ensure the inclusion of high-quality RCTs. Second, we did not assess cardiopulmonary function owing to the lack of data in multiple studies. In the future, cardiopulmonary function in CHD patients who accept Baduanjin exercise should be analyzed. Third, because of the lack of hard end points, such as the rate of rehospitalization or mortality, more RCTs involving hard outcomes should be conducted in the future. Therefore, this study requires further improvement in the future because we did not analyze the effects of Baduanjin exercise with different intensities, times, and frequencies on cardiac function, quality of life and psychological status of CHD patients after PCI. In addition, the literature on Baduanjin exercise therapy is limited, and the total sample size is relatively small. Hence, high-quality RCTs with a larger sample size are needed to confirm and generalize the results.

## 5. Conclusions

Baduanjin exercise was safe and effective in improving cardiac function and quality of life in patients with CHD after PCI, and should be used as a complementary and alternative therapy for CR to treat CHD and other related chronic diseases.

## Author contributions

**Conceptualization:** Xingxing Li, Zongjing Fan.

**Funding acquisition:** Yang Wu, Zongjing Fan.

**Methodology:** Quan Lin, Rongpeng Liu, Yang Wu.

**Project administration:** Quan Lin, Zongjing Fan.

**Writing – original draft:** Xingxing Li.

**Writing – review & editing:** Xingxing Li, Zongjing Fan.
